# Presence of proNGF-Sortilin Signaling Complex in Nigral Dopamine Neurons and Its Variation in Relation to Aging, Lactacystin and 6-OHDA Insults

**DOI:** 10.3390/ijms140714085

**Published:** 2013-07-08

**Authors:** Yi Xia, Bei-Yu Chen, Xiao-Long Sun, Li Duan, Guo-Dong Gao, Jing-Jie Wang, Ken Kam-Lin Yung, Liang-Wei Chen

**Affiliations:** 1 Institute of Neurosciences, Fourth Military Medical University, Xi’an 710032, China; E-Mails: shylockk-xy@hotmail.com (Y.X.); fmmulong@foxmail.com (X.-L.S.); duanduanli@hotmail.com (L.D.); 2 Department of Neurosurgery, Tangdou Hospital, Fourth Military Medical University, Xi’an 710038, China; E-Mail: gaogdneurosurgery@126.com; 3 Department of Orthopedics, Xijing Hospital, Fourth Military Medical University, Xi’an 710032, China; E-Mail: chenby@fmmu.edu.cn; 4 Department of Gastroenterology, Tangdou Hospital, Fourth Military Medical University, Xi’an 710038, China; 5 Department of Biology, Baptist University of Hong Kong, Hong Kong, China; E-Mail: kklyung@hkbu.edu.hk

**Keywords:** pro-neurotrophins, pro-neurotrophin receptors, neurodegeneration, Parkinson’s disease

## Abstract

Growing evidence has shown that proNGF-p75NTR-sortilin signaling might be a crucial factor in neurodegeneration, but it remains unclear if it may function in nigral neurons under aging and disease. The purpose of this study is to examine and quantify proNGF and sortilin expression in the substantia nigra and dynamic changes of aging in lactacystin and 6-hydroxydopamine (6-OHDA) rat models of Parkinson’s disease using immunofluorescence, electronic microscopy, western blot and FLIVO staining methods. The expression of proNGF and sortilin was abundantly and selectively identified in tyrosine hydroxylase (TH)-containing dopamine neurons in the substantia nigra. These proNGF/TH, sortilin/TH-positive neurons were densely distributed in the ventral tier, while they were less distributed in the dorsal tier, where calbindin-D28K-containing neurons were numerously located. A correlated decrease of proNGF, sortilin and TH was also detected during animal aging process. While increase of proNGF, sortilin and cleaved (active) caspase-3 expression was found in the lactacystin model, dynamic proNGF and sortilin changes along with dopamine neuronal loss were demonstrated in the substantia nigra of both the lactacystin and 6-OHDA models. This study has thus revealed the presence of the proNGF-sortilin signaling complex in nigral dopamine neurons and its response to aging, lactacystin and 6-OHDA insults, suggesting that it might contribute to neuronal apoptosis or neurodegeneration during pathogenesis and disease progression of Parkinson’s disease; the underlying mechanism and key signaling pathways involved warrant further investigation.

## 1. Introduction

Parkinson’s disease (PD) is a severe debilitating and neurodegenerative disease in human beings that is characterized by motor symptoms of tremor, bradykinesia, rigidity and postural instability. Since it results from the progressive death of dopamine neurons in the substantia nigra and pharmacological levodopa intervention to elevate dopamine alleviates the patient symptoms but cannot halt disease progression, neurotrophic therapy is widely recommended for PD treatment [1–3]. Dopamine neurons, particularly those located in the ventral tier group of substantia nigra pars compacta with their dendrite extension ventrally into the substantia nigra pars reticularis (and biochemically calbindin-D28K negative), showed selective neuronal cell death or high susceptibility to various neurotoxin insults, e.g., 1-methyl-4-phenyl-1,2,3,6-tetrahydropyridine (MPTP), 6-hydroxydopamine (6-OHDA), or lactacystin that were used to induce PD animal models [4–6]. Until now, the selective death mechanism has remained obscure and a serious obstacle in clinical cure of the disease. Growing evidence has suggested a role for proNGF-p75NTR-sortilin signaling in neurodegeneration and pathogenesis of PD [7], and we have identified the p75 neurotrophin receptor (p75NTR) in the dopamine neurons and kainic acid-induced up-regulation of p75NTR accompanying neuronal degeneration in the substantia nigra [8]. Studies from other authors also indicated some functions of proNGF, p75NTR, and sortilin in regulating neuronal cell survival, apoptosis and growth cone collapse in aging and pathological events [9–12]. In addition, abnormality in neurotrophic support or deficiency in neurotrophic factor might be significant contributing factors in neurodegeneration, pathogenesis and progression of PD [2,7].

Moreover, recent studies have revealed that p75NTR may act as “molecular signaling switch” that determines neuronal cell survival or neuronal cell death [10,13]. The proNGF triggers neuronal cell apoptosis by high-affinity binding to p75NTR, while p75NTR mediates neuronal cell death by formation of the p75NTR-sortilin signaling complex [9,12,13]. In fact, proNGF, p75NTR and sortilin exhibited roles in neurodegeneration of aging, onset or progression in various neurodegenerative diseases like Alzheimer’s disease (AD), amyotrophic lateral sclerosis (ALS) and acute trauma of the nervous system [14–19]. Currently, however, there is still a lack of studies on the implication of proNGF-p75NTR-sortilin signaling in pathogenesis or progression of PD. Based on previous evidence, we hypothesize that proNGF-p75NTR-sortilin signaling may possibly contribute to the vulnerability of nigral neurons. This research study was thus conducted in aging, 6-OHDA and lactacystin animal models by using immunohistochemistry, laser scanning confocal microscopy, electron microscopy, western blot and FLIVO staining methods. Results of this study have presented novel proNGF-sortilin signaling complex specifically assembled in dopamine neurons of substantia nigra, which might be possibly involved in neuronal apoptotic and degenerative death subject to brain aging, 6-OHDA and lactacystin insults.

## 2. Results and Discussion

### 2.1. Identification of Abundant proNGF in A9 Ventral Tier Dopamine Neurons of the Substantia Nigra

**Figure 1:**
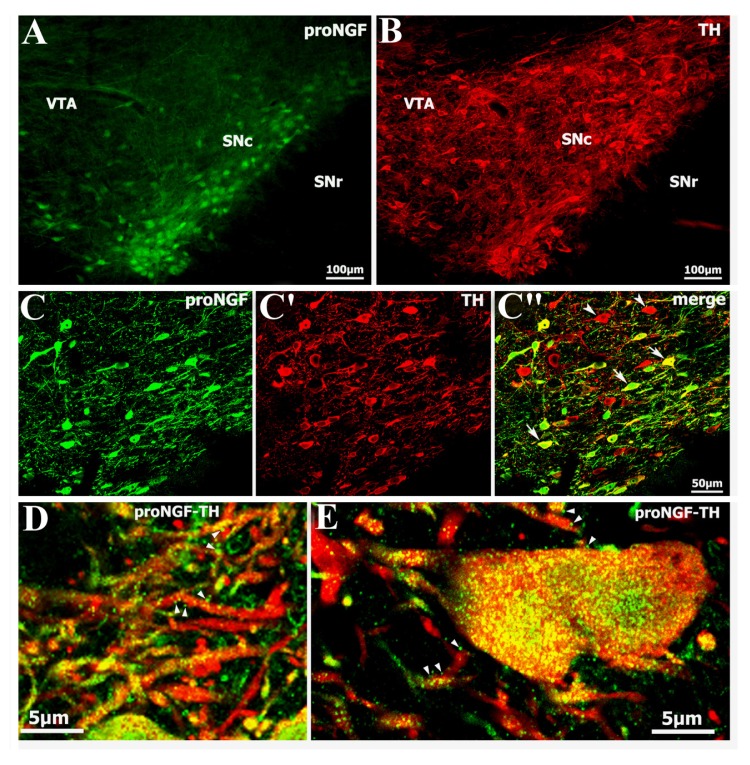
Laser scanning confocal microscopy showing localization of proNGF in dopamine neurons of the substantia nigra of adult rats. The proNGF/TH double-labeled cells are densely distributed in the A9 ventral tier of the substantia nigra pars compacta (**A**–**C**); The proNGF-positive punctuates or granules (green or yellow color) are localized in neuronal cytoplasm, membrane and extracellular matrix (**D**,**E**). Arrows indicate proNGF/TH double-labeled neurons (**C″**), while arrowheads indicate TH single-labeled neurons (**C″**) or show proNGF-positive punctuates (**D**,**E**). Abbreviations: SNc, substantia nigra pars compacta; SNr, substantia nigra pars reticularis; VTA, ventral tegemental area.

Immunoreactivity to proNGF was clearly identified in the dopamine neurons of midbrain sections of adult rats. The proNGF/TH-positive neurons were densely distributed in the ventral tier of the substantia nigra pars compacta (SNc), while they were scarce in the substantia nigra pars lateralis (SNr) and ventral tegemental area (VTA) (Figure 1A–C). Under higher magnification confocal microscopy, proNGF-positive punctuates were seen within neuronal cytoplasm, attached to neuronal dendrites or distributed in the extracellular matrix (Figure 1D,E). The specificity of the proNGF antibody used for immunohistochemistry was confirmed by substitution and adsorption control experiments (data not shown). Cell count data indicated that proNGF/TH double-labeled neurons constituted about 68% of total TH-positive cells in the substantia nigra.

**Figure 2:**
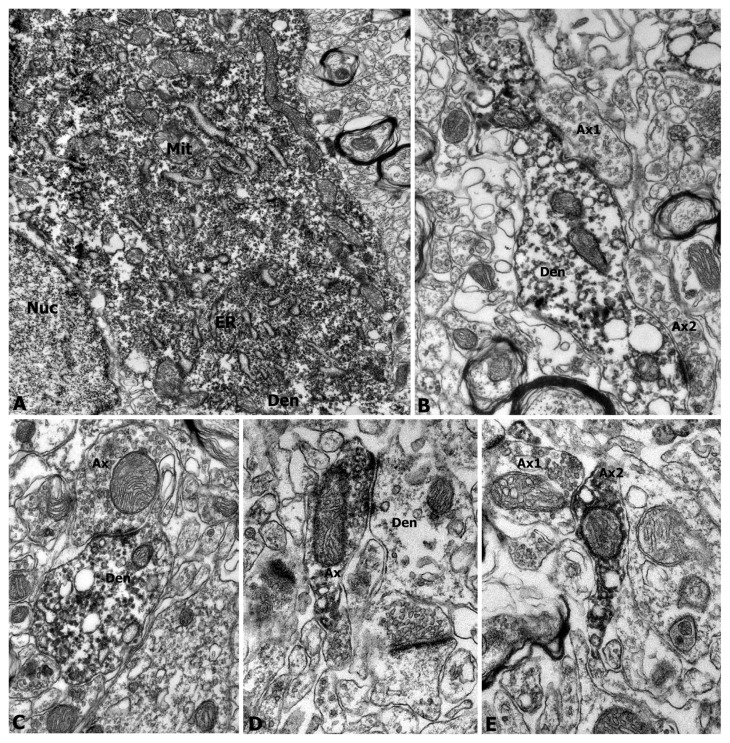
Electron microscopy showing proNGF-positive neuronal somas, dendrite and axonal terminals in nigral and striatal regions of adult rats. The proNGF-positive neuronal somas and dendrites in the substantia nigra (**A**–**C**) and proNGF-positive axonal terminals in the striatum (**D**,**E**) are representatively showed. The proNGF-positive products with high density are observed in soma (**A**), dendrites (**B**,**C**), and axonal varicosities or terminals with synapses or synaptic buttons (**D**,**E**). Abbreviations: Ax, axon; Den, dendrite; ER, endoplasmic reticulum; Mit, mitochondria; Nuc, nucleus.

Immunoelectron microscopy was further applied to show ultrastructure of proNGF-containing neurons in nigra and striatum of adult rats. Dense proNGF-immunoreactivity was observed in neuronal somas, dendrites and axonal terminals of the nigral neurons (Figure 2). The proNGF-positive products were densely located in the endoplasmic reticulum, vesicles, mitochondrial membrane and cytoplasm. These proNGF-positive soma (Figure 2A) and dendrites (Figure 2B,C) were clearly shown in the substantia nigra, while proNGF-positive axons or axonal terminals were numerously detected in the striatum (Figure 2D,E). The proNGF-positive dendrites received synaptic buttons from axonal terminals in the substantia nigra (Figure 2B,C), while these proNGF-positive axon terminals made synaptic contacts on dendrites in the striatum (Figure 2D,E).

**Figure 3:**
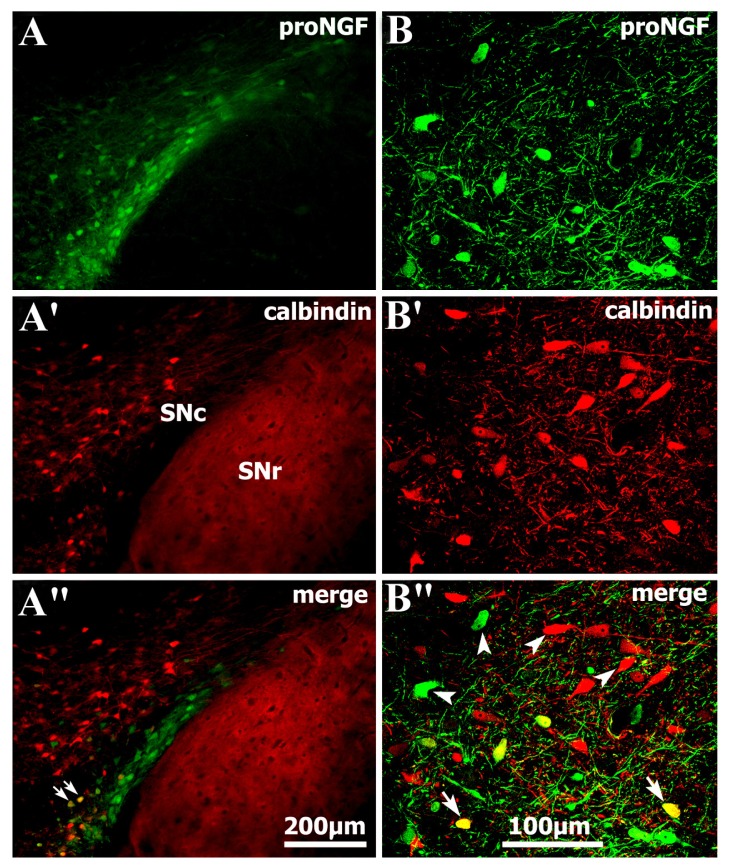
Double immunofluorescence showing colocalization of proNGF and calbindin-D28K in the nigral neurons. (**A-A″**) Only are a few of proNGF/calbindin-D28K double-labeled neurons detected in the ventral tier group of substantia nigra, and double-labeled ones are indicated with arrows; and (**B-B″**) while more single-labeled ones are representatively indicated with arrowheads, which are shown with higher magnification in the right column. Abbreviations: SNc, substantia nigra pars compacta; SNr, substantia nigra pars reticularis.

To examine the properties of proNGF-positive neurons, proNGF/calbindin-D28K double labeling was performed to assess whether proNGF-positive neurons overlap calbindin-D28K-containing neurons in the substantia nigra and the VTA. Distribution of proNGF-positive neurons in the ventral tier of SNc scarcely overlapped calbindin-D28K-positive neurons, whereas calbindin-D28K-positive ones were mainly distributed in the dorsal tier of the SNc and VTA (Figure 3A–A″). Nevertheless, a few of the scarce proNGF/calbindin-D28K double-labeled neurons were still detected in the ventral tier group of SNc region and shown with high magnification (Figure 3B–B″).

### 2.2. Selective Distribution of proNGF/TH Neurons in Substantia Nigra and Age-Related Changes

**Figure 4:**
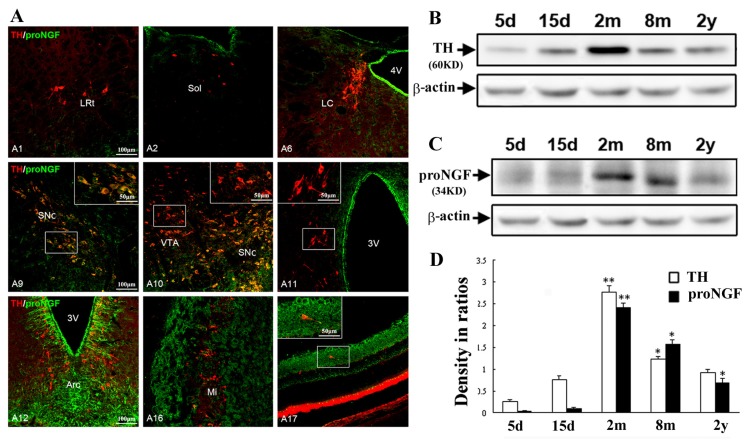
Selective distribution of proNGF/TH neurons and age-related changes in the substantia nigra. (**A**) Distribution patterns of proNGF/TH-positive neurons in all A1–A17 cell groups of whole rat brains. Immunoreactive cells are shown in A1 (lateral reticular area of medulla), A2 (nucleus of solitary tract), A6 (locus coeruleus), A9 (substantia nigra), A10 (ventral tegemental area), A11 (periventricular hypothamic nucleus), A12 (arcuate hypothalamic nucleus), A16 (olfactory bulb) and A17 (retina) areas; (**B**) Immunoblotting of proNGF expression in substantia nigra of postnatal 5 days, 15 days, 2 months, 8 months and 2 years old; (**C**) Immunoblotting of TH expression in substantia nigra of postnatal 5 days, 15 days, 2 months, 8 months and 2 years old; and (**D**) Comparison of proNGF and TH expression levels in the substantia nigra in ratio to internal control β-actin. ANOVA indicates significance, *p < 0.005, **p < 0.001 *vs.* earlier time-point of animals.

Double immunofluorescence was performed to determine if proNGF/TH neurons were selectively distributed in the substantia nigra of all A1–A17 cell groups including medulla (A1–A3), pons (A4–A7), midbrain (A8–A10), hypothalamus (A11–A15), olfactory bulb (A16) and retina (A17). It was revealed that proNGF/TH double-labeled neurons were predominately located in the A9 ventral tier group of the substantia nigra, scarcely observed in A1 medulla and A17 retina regions, and they were not detected in A11–A15 hypothalamic and A16 olfactory bulb regions where numerous proNGF-positive cells or structures were also distributed (Figure 4A). In the locus coeruleus, however, proNGF-positive axonal processes were also distributed among TH-positive somas and some of them contacted TH-positive somas. In addition, numerous proNGF-positive ependymal cells were densely observed in the forebrain, diencephalons and brain stem as well.

Western blot analysis was further performed to show aging changes of proNGF and TH expression in the substantia nigra at 5 days, 15 days, 2 months, 8 months and 2 years old. Expression of proNGF and TH in the substantia nigra was weak at 5 days and 15 days (developing stage), went up and reached the highest level at 2 months (adult stage), and gradually went down at 8 months and 2 years (aging stage) (Figure 4B,C). Interestingly, in aging animals, dying neurons were consistently observed in the substantia nigra by Fluoro-Jade staining that could visualize neuronal degeneration (data not shown). Quantitative analysis indicated that significant decreases of proNGF and TH protein expression levels occurred along with neuronal loss in the substantia nigra of aging rats with 8 months and 2 years old (Figure 4D).

### 2.3. Abundant Distribution of Sortilin/TH Neurons in Substantia Nigra and Age-Related Changes

**Figure 5:**
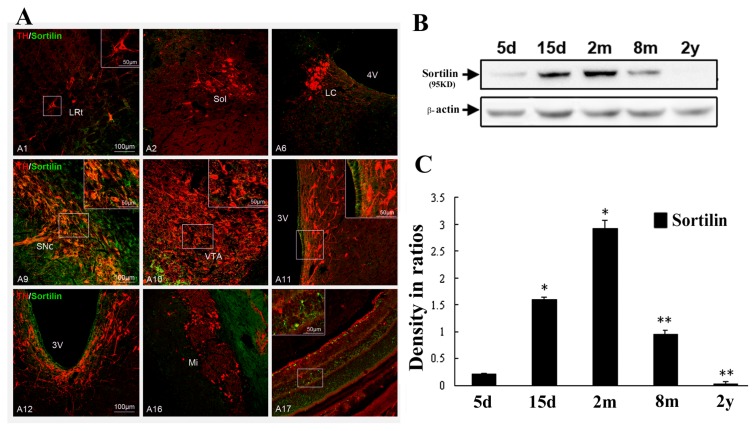
Abundant distribution of sortilin/TH neurons and age-related changes in the substantia nigra. (**A**) Distribution patterns of TH/sortilin-positive neurons in all A1–A17 cell groups of whole rat brains. TH-, sortilin-, and TH/sortilin-positive neurons are shown in A1, A2, A6, A9, A10, A11, A12, A16 and A17 areas, representatively; (**B**) Immunoblotting of sortilin expression in the substantia nigra of postnatal 5 days, 15 days, 2 months, 8 months and 2 years old; and (**C**) Comparison of sortilin expression levels in the substantia nigra in ratio to β-actin. ANOVA test indicates significance, *p < 0.005, **p < 0.001 *vs.* earlier time-points.

Double immunofluorescence was also carried out to examine if sortilin-positive neurons were selectively distributed in the substantia nigra of all A1–A17 cell groups in whole brains. It revealed that sortilin/TH double-labeled cells were predominate in the A9 ventral tier dopamine group of the substantia nigra, scarcely observed in A1 medulla and A17 retina regions, and they were not detected in A11–A15 hypothalamic and A16 olfactory bulb areas where sortilin-positive cells were also distributed (Figure 5A). In a distribution pattern similar to proNGF, sortilin-immunoreactivity was also densely observed in the ependymal cells in the forebrain, diencephalons and brain stem regions. Cell count data indicated that the sortilin/TH double-labeled neurons constituted about 73% of total TH-positive ones in the SNc region. Additionally, double immunofluorescence showed a high level (about 86%) of colocalization of proNGF and sortilin in these nigral neurons (data not shown).

Western blot analysis was also performed to show aging dependent changes of sortilin expression levels in the substantia nigra at 5 days, 15 days, 2 months, 8 months and 2 years old. It revealed that expression of sortilin in the substantia nigra was weak at 5 days, went up at 15 days, reached the top level at 2 months, and went down at 8 months and 2 years old, which was consistent with the expression pattern of proNGF changes (Figure 5B). Quantitative analysis of data indicated that significant decreases of sortilin expression also occurred in aging rats at 8 months and 2 years (Figure 5C), which was consistent with the occurence of dopamine neuronal degeneration visualized by Fluoro-Jade staining in the substantia nigra of aging animals.

### 2.4. Neurodegenerative or Dying Sensitivity of proNGF-Containing Neurons to Lactacystin Insult

**Figure 6:**
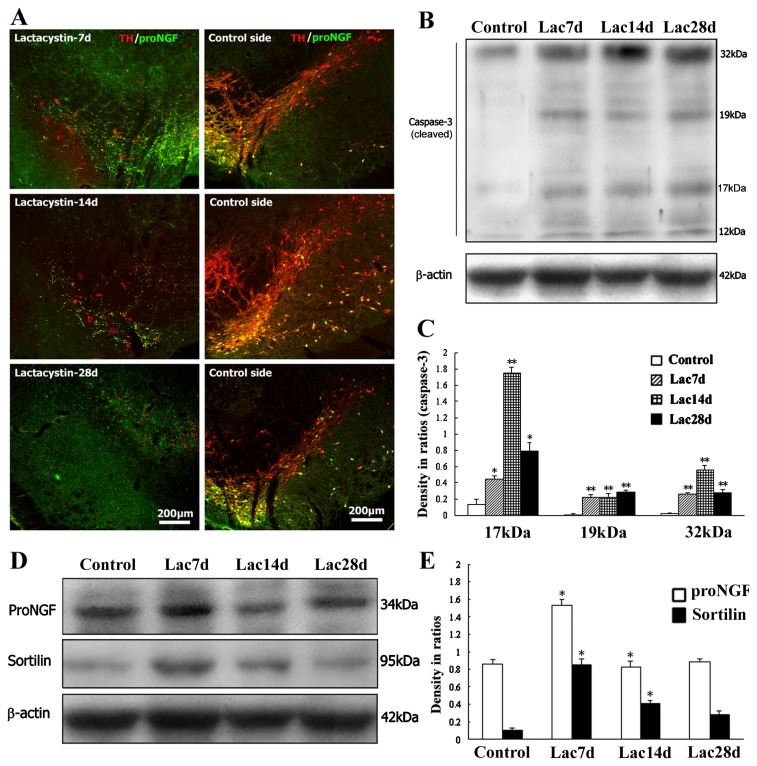
The proNGF-containing dopamine neurons in the substantia nigra show vulnerability to lactacystin insult. (**A**) The proNGF/TH double-labeled neurons exist numerously in the control nigral side, while they died off mostly at 7 days, or completely at 14 days and 28 days in the lesioned side; (**B**) Immunoblotting of active caspase-3 in the substantia nigra of the lactacystin model; (**C**) Comparison of cleaved caspase-3 (17 kDa, 19 kDa, 32 kDa) expression among control, lac7 days, lac14 days and lac28 days; (**D**) Immunoblotting of proNGF and sortilin in the substantia nigra of rat lactacystin model; and (**E**) Comparison of proNGF and sortilin expression levels among control, lac7 days, lac14 days and lac28 days. ANOVA test: *p < 0.005, **p < 0.001, *vs.* control or earlier time-point of lactacystin model.

Moreover, in a rat model with a unilateral lactacystin lesion, abnormal changes in proNGF and TH-positive neurons were also observed by a comparison of normal and lesioned nigra. The proNGF or TH-positive dopamine neurons mostly died at 7 days, and completely died at 14 days or 28 days after lactacystin lesion. Some proNGF or TH-positive neuronal processes were still detected at time-points of 7 days and 14 days, whereas almost all or all proNGF or TH-positive neuronal somas disappeared. In addition, the proNGF-positive glial-like cells were observed in nigra and remarkably increased their numbers in the substantia nigra pars compacta and reticularis at 28 days following lactacystin insult (Figure 6A).

Western blot analysis was further performed to detect changes of proNGF, sortilin and cleaved (active) caspase-3 in the lactacytsin model at 7 days, 14 days and 28 days. Dynamic change patterns of proNGF, sortilin and caspase-3 expression were observed in the substantia nigra after lactacystin insult. By densitometry of immunoblots in related ratio to internal control β-actin levels abnormal increases of both proNGF and sortilin proteins in lesioned nigra were revealed in comparison with that of controls, which exhibited a correlated increase of cleaved caspase-3 (17, 19 and 32 kDa) expression levels (Figure 6B–E).

### 2.5. Neurodegenerative Vulnerability of proNGF-Containing Neurons to 6-OHDA Insult

**Figure 7:**
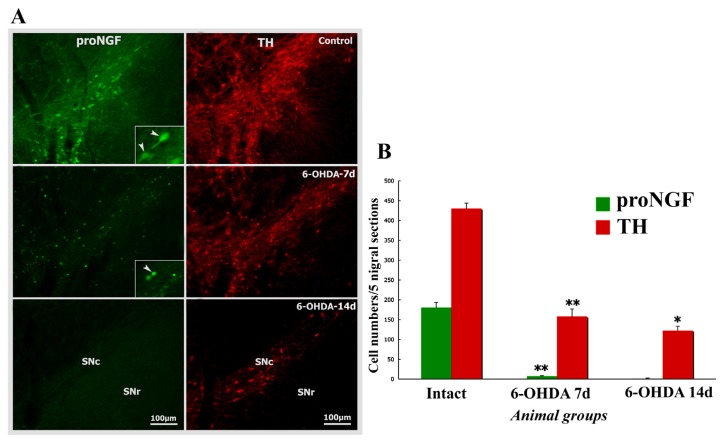
The proNGF-containing dopamine neurons in the substantia nigra show their vulnerability to 6-OHDA insult. (**A**) The proNGF/TH double-labeled neurons exist numerously in control, died off mostly at 7 days or completely 14 days, while TH single-labeled neurons still remain in the lesion side of the substantia nigra at 7 days or 14 days after 6-OHDA insult; and (**B**) Comparison of proNGF or TH-positive neuronal cells among control, 6-OHDA-7d and 6-OHDA-14d. ANOVA test: *p < 005, **p < 001, *vs.* control or earlier time-point of 6-OHDA model.

In a 6-OHDA unilateral lesion rat model, proNGF and TH-positive neurons were also examined by the comparison of normal and lesioned nigra. The proNGF-positive dopamine neurons mostly died at 7 days, and completely died at 14 days following 6-OHDA lesion. However, the TH-positive neurons still remained in the lesion side of SNc where proNGF-positive neurons were not detected at 14 days following 6-OHDA insult (Figure 7A). Cell count data showed that proNGF-positive neuronal cells in lesion side of SNc underwent a dynamic and fast cell death after receiving unilateral 6-OHDA injections (Figure 7B).

**Figure 8:**
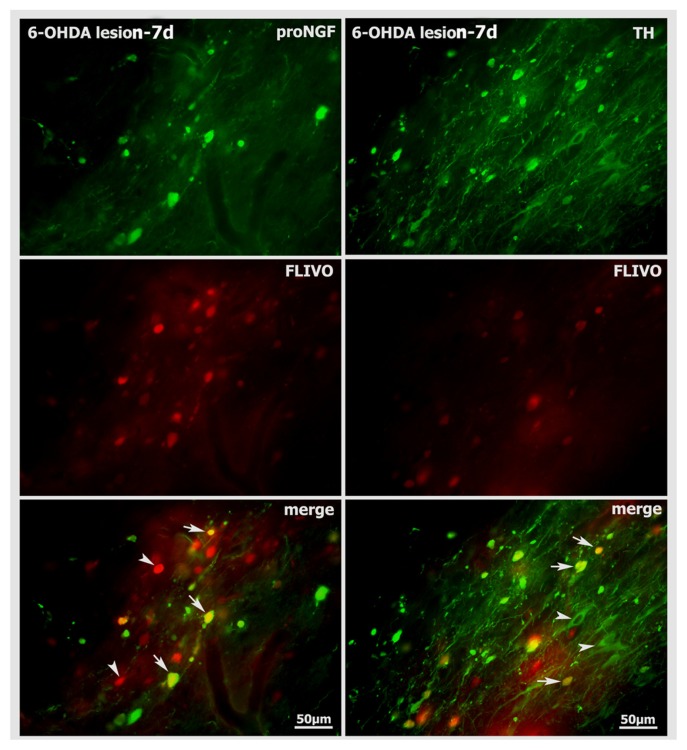
FLIVO staining shows neuronal cell death in the substantia nigra of 6-OHDA rat models. In the left column, proNGF-positive (green color), FLIVO-positive (red color, arrowheads) and FLIVO/TH double-labeled (yellow color, arrows) cells are shown. In the right column, TH-positive (green color, arrowheads), FLIVO-positive (red color) and FLIVO/TH double-labeled (yellow color, arrows) cells are shown.

FLIVO staining, which can specifically stain apoptotic cells *in vivo* [20], was also performed to show proNGF-positive or TH-positive neuronal cells under apoptosis in the nigra of 6-OHDA lesion at time-point of 7 days. FLIVO-positive cells were clearly detected in lesioned nigra, and some of them were overlapped with proNGF or TH-immunoreactive neurons in their distribution (Figure 8). Cell count data indicated that about 54% of FLIVO-positive cells were proNGF-containing neurons, while 88% of FLIVO-positive cells were TH-immunoreactive neurons in lesion nigra side.

A new finding of the study is the identification of the presence of a novel proNGF-sortilin signaling complex in the ventral tier dopamine neurons of substantia nigra, which appeared to be involved in neuronal vulnerability or neuronal death to aging, lactacystin and 6-OHDA insults. Briefly, proNGF was abundantly observed in ventral tier neurons and characterized with low calbindin colocalization and high sortilin coexpression rate. The proNGF/TH-positive neurons were selectively distributed in the substantia nigra (A9 group) of whole A1–A17 cell populations. Correlated dynamic changes of proNGF, sortilin and TH were found in aging, lactacystin and 6-OHDA rat models of PD. This study has, overall, indicated that the proNGF-sortilin signaling compex assembled in substantia nigra might possibly play a role in neuronal vulnerability and contribute to neurodegeneration, pathogenesis and disease progression of PD [7,10,14,17,21].

Growing attention is being paid to the unexpected and opposing, or double-edge effects of various neurotrohins in functional development and pathological implications in neurological diseases [13,22]. Neurotrophins such as NGF, BDNF and NT3 are initially synthesized as proforms of neurotrophins, *i.e.*, proNGF, proBDNF and proNT3, followed by cleavage to release *C*-terminal mature forms in response to physiological changes. Mature neurotrophins are the preferred ligands for Trks and signals emanated from Trks support cell survival, neurite growth and synaptic strengthening of neurons, while these pro-neurotrophins bind preferentially to p75NTR-sortillin complex and signals emanated from p75NTR-sortilin induce cell apoptosis, growth cone collapse and synaptic weakening of central neurons [12,13,23,24]. Neurotoxic and neurotrophic roles of proNGF and the receptor sortilin were observed in the adult and aging nervous system, and proNGF showed effects on both neuronal viability and neurite growth [22,25]. By high-affinity binding to p75NTR and sortilin complex, proNGF mediated cell death of central neurons, natural killer cells and retina photoreceptors [26–28]. Further, proNGF showed an inhibitory effect on NGF-mediated TrkA activation in PC12 cells [29]. ProNGF also induced PTEN via p75NTR to suppress Trk-mediated survival signaling in central neurons [30]. The p75NTR mediated neuronal cell death by activating GIRK channels through phosphatidylinositol 4,5-bisphosphate [31]. Apoptosis induced by p75NTR overexpression required Jun kinase-dependent phosphorylation of Bad, and neurotrophin receptor interacting factor was also an essential mediator of apoptotic signaling induced by p75NTR [32,33]. In addition, p75NTR-induced autophagy was also identified in cerebellar Purkinje neurons [34]. Consistently, new data in this study indicated correlation of abnormal changes of proNGF-sortilin molecules with neuronal apoptosis or degenerative death in the substantia nigra of aging, 6-OHDA and lactacystin animal models.

Moreover, proNGF-p75NTR-sortilin signaling might be initiated by released pro-neurotrophins from glial cells including microglias and astrocytes. While reactive microglias and astrocytes might constitute major sources of released pro-neurotrophins under injury and pathological events [4] neuronal cell survival or death might be critically regulated by secreted pro-neurotrophins [35]. Microglia-derived proNGF also promoted photoreceptor cell death [36]. Application of microglial inhibitor minocycline alleviated death of oligodendrocytes by inhibiting proNGF production in microglial cells in spinal cord injury [37]. Astrocytic production of NGF was implicated in motor neuronal apoptosis of ALS animal models [38]. Neurotrophic actions initiated by proNGF in adult sensory neurons required peri-somatic glia to drive cleavage to NGF [39]. Besides, proNT3, proBDNF, and toxic extracellular Abeta also induced neuronal apoptosis by the receptor complex of p75NTR and sortilin [40–42]. The reactive glial cells that were commonly detected in excitotoxic, traumatic and ischemic brains [38], predominately surrounded the lesion neurons in AD and ALS, and were abundantly distributed in nigrostriatal regions of PD models [4], implying that reactive glia cells are implicated in neuronal degenerative loss or disease progression via releasing pro-neurotrophins and neuronal-glial interaction in ALS, AD, and PD [38].

Finally, proNGF-p75NTR-sortilin signaling cascades might be significantly involved in aging, brain injury, disease onset and progression of neurodegenerative diseases [10,31,43]. For instance, age-dependent alterations in proNGF, sortilin proteins and age-related neurodegeneration were correlated in several types of neurons [14,44,45]. Increased proNGF, sortilin and p75NTR also acted as mediators of injury and ischemia-induced neuronal apoptosis or degeneration [15,18,46,47]. The proNGF/NGF imbalance was demonstrated to link vulnerability of cholinergic neurons, neuronal degeneration and onset of AD [16,17,48–50]. Diabetes-induced imbalance of proNGF/NGF might constitute one cause of neurovascular injury [51]. Up-regulation of proNGF, p75NTR and sortilin was also associated with spongiform encephalomyelopathy [52]. Reduced p75NTR delayed disease onset in female transgenic ALS mice [19], though treatment with a p75NTR antagonist showed no improvement on disease progression [53]. Furthermore, while the lactacystin and 6-OHDA rat models of PD were used in this study, previous evidence showed that they might differ in their mechanism to induce neuronal death; lactacystin might inhibit proteasomes and induce modified protein, and 6-OHDA might function through oxidative stress and induce mitochondrial dysfunction [1,5,6]. In a lactacystin rat model of this study, an increase of proNGF and sortilin expression levels appeared to be related to cleaved caspase-3 expression in the substantia nigra. The increasing expression of p75NTR was also identified in nigral dopamine neurons and neurodegeneration induced by kainic acid insult in our previous study [8]. In a 6-OHDA rat model of this study, we found that proNGF-positive dopamine neurons were sensitive and highly susceptible to death after 6-OHDA insult in the substantia nigra. In addition, Wang *et al.* also reported involvement of proNGF-p75NTR-sortilin signaling in cell apoptosis in a 6-OHDA rat model; their study revealed an increase of proNGF, p75NTR and sortilin expression levels, a decrease of NGF and TrkA expression, and activation of a caspase-related signal pathway in the cell death of dopamine neurons in the substantia nigra [54]. Obviously, the balance between the signal pathways activated by the proNGF-p75NTR-sortilin and the NGF-TrkA system might be critical to determine the cell death or cell survival of neurons [7,54]. These data together imply that the proNGF-p75NTR-sortilin signaling complex might be involved in neuronal cell death or apoptosis of substantia nigra of the PD model [7,8,54]. This study has provided evidence of the relation of proNGF-sortilin complex with aging, lactacystin and 6-OHDA animal models. Therefore, it suggests a possible important role of the proNGF-p75NTR-sortilin signaling complex in pathogenesis or disease progression of PD, which shall need further investigation by gene knock-down or knock-out of the proNGF-p75NTR-sortilin complex. Therefore, a further understanding of proNGF-p75NTR-sortilin signaling in neuron-glial interaction and neurological disorders shall provide targets for the treatment of PD [2,3,21,55].

## 3. Experimental Section

### 3.1. Animals and Animal Models

Ninety-two *Sprague-Dawley* rats were used in total for this study and supplied from the Animal Center of the Fourth Military Medical University (FMMU), China. These rats included the postnatal aging group: 5 days (*n* = 8), 15 days (*n* = 8), 2 months (*n* = 8), 8 months (*n* = 8) and 2 years (*n* = 8); 6-OHDA lesion group: control (*n* = 6), lesion 7 days (*n* = 10), 14 days (*n* = 6) and 28 days (*n* = 6); lactacystin lesion group: control (*n* = 6), lesion 7 days (*n* = 6), 14 days (*n* = 6) and 28 days (*n* = 6). All animal experiments were carried out in according with the National Institute of Health guide for the care and use of Laboratory animals (NIH Publications No. 80-23) revised 1996, approved by the Committee of Animal Use for Research and Education of FMMU, and all efforts were made to minimize animal suffering and reduce the number of animals used.

For preparation of the 6-OHDA model, rats received unilateral injections of 3 μL 6-OHDA solution (0.01 mg in 3 μL saline containing 0.2 mg/mL ascorbic acid) into the right medial forebrain bundle and were allowed to survive for 7 days, 14 days and 28 days in reference to previous report [5]. Similarly, for preparation of lactacystin model, rats received injections of 3 μL lactacystin solution (10 μg in 3 μL saline) in the right medial forebrain bundle, and saline injection was used in the controls. These animals were allowed to survive for 7 days, 14 days and 28 days after 6-OHDA or lactacystin surgical injection, thereafter used for immunohistochemistry and western blotting experiments, respectively.

### 3.2. Brain Tissue Preparation

For sample preparation for immunohistochemstry, these animals included postnatal aging groups: 7 days (*n* = 5), 14 days (*n* = 5), 2 months (*n* = 5), 8 months (*n* = 5) and 2 years (*n* = 5); 6-OHDA lesion group: control (*n* = 3), lesion 7 days, 14 days and 28 days (*n* = 9); lactacystin lesion group: control (*n* = 3), lesion 7 days, 14 days and 28 days (*n* = 9), and were deeply anesthetized with sodium pentobarbital (40 mg/kg, i.p.) and perfused transcardially with 4% paraformaldehyde in 0.1 M (pH 7.4). The brains and retinas (containing A1–A17 cell groups) were removed immediately and placed in 0.1 M PB containing 30% sucrose overnight at 4 °C. After, the brain and retina samples were serially cut into coronal sections (30 μm) on a frozen microtome and rinsed in 0.01 M phosphate buffered saline (PBS, pH 7.4), and retina sections were mounted on glass-slides for immunohistochemistry. The brain samples of remaining animals were utilized for western blot experiments.

### 3.3. Immunofluorescence

Double immunofluorescence and laser scanning confocal microscopy (LSCM) were performed to reveal localization of proNGF and sortilin in TH-containing neurons in whole brains and retinas of above animals. Double staining with proNGF/TH, sortilin/TH, or proNGF/calbindin-D28K was performed respectively. Briefly, the sections were incubated with 10% donkey serum-containing blocking solution for 30 min at room temperature, and followed by incubation of primary antibody solution containing 10% donkey serum, 0.1% triton X-100 in PBS at 4 °C for 24 h, *i.e.*, rabbit anti-proNGF (P5498, Sigma-aldrich, St Louis, MO, USA), rabbit anti-sortilin (S0697, Sigma-aldrich), mouse anti-calbindin-D28K (C9848, Sigma-aldrich), mouse anti tyrosine hydroxylase (T1299, Sigma-aldrich), respectively. After three washes with PBS, the sections were further incubated with Alexa Fluor-488, or Alexa Fluor-594 conjugated donkey anti-mouse or rabbit IgG (Molecular Probes) for 4 h at room temperature. The sections were mounted with Fluorescence-preserving VECTASHIELD Mounting medium (Vector, H-1000, Vectorlabs, Burlingame, CA, USA) and examined under LSCM (FV1000, Olympus, Tokyo, Japan). In addition, for immunostaining control experiments, primary antibody was substituted with normal mouse or rabbit serum and by adsorption control confirmation. The immunoreactive cells were not detected in these control staining samples.

### 3.4. Immunoelectron Microscopy

To enhance the tissue penetration of antibodies for electron microscopy study, the vibrate sections were equilibrated in a cryoprotectant solution (0.05 MPB, pH 7.4, containing 25% sucrose and 10% glycerol) for at least 3 h. The sections were then freeze-thawed by freezing in isopentane cooled in liquid nitrogen, followed by liquid nitrogen, and thawing in PBS. The sections were then washed several times in PBS and incubated in 10% normal donkey serum in PBS for 1 h. All incubation steps were carried out in PBS containing 1% normal donkey serum and sections were washed three times between steps. For immunoperoxidase, sections were incubated with rabbit anti-proNGF or mouse anti TH for 48 h at 4 °C. The sections were then incubated in biotin-conjugated donkey anti-rabbit IgG or donkey anti-mouse IgG (Vector) for 12 h at 4 °C, followed by avidin-biotin-peroxidase complex (ABC; Vector) for 4 h at room temperature. After equilibrating in Tris buffer (0.05 M, pH 7.6), the peroxidase was visualized by incubation in 0.025% diaminobenzidine (Sigma-aldrich) in Tris buffer in the presence of 0.01% H_2_O_2_. The reaction was stopped after 8–10 min by three washes in Tris buffer.

Processing for electron microscopy was performed in following steps. All sections were washed three times in PB and post-fixed with 1% osmium tetroxide for 20 min. After PB washes, the sections were dehydrated through a graded series of dilutions of ethanol, with 1% uranyl acetate added to the 70% ethanol solution. Following absolute ethanol, sections were treated with propylene oxide (Sigma-aldrich) and placed in resin overnight. The sections were then mounted on glass slides, a coverslip applied and the slides were placed in an oven at 60 °C for 48 h. After examination in the light microscope, regions of interest within the substantia nigra were cut out and glued onto resin blocks. Serial ultrathin sections (approximately 70 nm) were cut on a Riechert-Jung Ultracut E ultramicrotome (Leica, Wetzlar, Germany) and collected on single-slot copper grids coated with pioloform. Ultrathin sections were contrasted with lead citrate for 3–4 min and examined under Philips CM 10 electron microscope, and selected images were taken for further analysis or demonstration.

### 3.5. Western Blot

Western blot was performed to quantify protein expression levels in midbrain samples in a standard protocol. Briefly, protein extracts were prepared from ventral midbrain tissues that were dissected from ventral midbrain containing substantia nigra. Fresh brain samples were homogenized at 4 °C in 5 volumes of extraction buffer [50 mM Tris (pH7.4), 150 mM NaCl, 1% NP-40, 0.5% sodium deoxycholate, 0.1% SDS, and protease inhibitor cocktail (Complete, Roche Diagnostics)], and the centrifugation of homogenates for 10 min (12,000× g) was performed. After measurement of total protein amount, supernatant was mixed with four volumes of protein loading buffer, boiled for 5 min at 99 °C and stored at 4 °C. Total protein (20–30 μg per loading passage) was loaded for electrophoresis on 10% denaturing PAGE gels and transferred to the nitrocellulose membrane (Bio-Rad). Membranes were blocked with 5% skimmed milk in Tris-buffered saline containing 0.05% Tween 20, then incubated with proNGF, sortilin, TH, or caspase-3 primary antibody, and followed by secondary antibody incubation and final visualization of bands (Bio-Rad). The rabbit anti-caspase-3 active (C8487, Sigma-aldrich) was used for immunoblotting. By using β-actin as internal control, quantitative analysis of immunoblotting bands was carried out and densitometry results in ratios were presented.

### 3.6. FLIVO Staining for Neuronal Degeneration

FLIVO staining method was applied to detect cell apoptosis of proNGF or TH-containing neurons in 6-OHDA rat models at 7 days (*n* = 4) by combining immunohistochemistry. FLIVO detection kit, a fluorescently labeled poly-caspase inhibitor for *in vivo* detection of apoptosis, can detect neuronal apoptosis in living brains of animals by systemic injection [20]. In this study, rats with unilateral 6-OHDA lesions at 7 days received FLIVO (20 μL diluted in 200 μL buffer (Immunochemistry Technologies, LLC, Waterloo, Australia) injection via tail vein and allowed to circulate for 1 h. After fixation and freezing, midbrain sections was followed by TH or proNGF immunofluorescence to determine if proNGF, TH-positive neurons undergo apoptosis. Dying or apoptotic cells stained by FLIVO and immunohistochemistry were visualized and captured in the substantia nigra under fluorescent microscopy.

### 3.7. Statistical Analysis

For quantitative data analysis, proNGF, sortilin, calbindin-D28K, TH and FLIVO-positive cells were counted in the substantia nigra and other groups of A1–A17 dopamine cell populations in brains. The numbers or percentages of double-stained cells, and density of immunoblotting bands in ratio to internal control β-actin were given as mean ± S.E.M. (*n* = 3–5). The differences between means were analyzed by one-way ANOVA (SPSS 18.0, Statistical Package for the Social Sciences). When ANOVA test showed significant difference among means, the pair-wise comparisons between means were also performed by *post hoc* testing. The significance level was set at a *p* value of less than 0.05 for all data analyses.

## 4. Conclusions

This study revealed the presence of the proNGF-sortilin signaling complex in the A9 ventral tier dopamine neuron group of substantia nigra, which appeared to be dynamic in expression levels and involved in nigral dopamine neuronal loss in aging, 6-OHDA and lactacystin rat models. Taken together with our previous observation, this study suggests that a novel proNGF-p75NTR-sortilin signaling complex might possibly contribute to neuronal vulnerability or neuronal death of dopamine neurons in the substantia nigra, and pathogenesis and disease progression of PD. Further investigation of underlying mechanisms shall benefit the development of new neuroprotection strategies by targeting proNGF-p75NTR-sortilin signaling cascades for the treatment of PD.

## Abbreviations

6-OHDA: 6-hydroxydopamine
AD: Alzheimer’s disease
ALS: amyotrophic lateral sclerosis
BDNF: brain-derived neurotrophic factor
MPTP: 1-methyl-4-phenyl-1,2,3,6-tetrahydropyridine
NGF: nerve growth factor
NT-3: neurotrophin-3
p75NTR: p75 neurotrophin receptor
PBS: phosphate buffered saline
PD: Parkinson’s disease
proBDNF: proform of brain-derived neurotrophic factor
proNGF: proform of nerve growth factor
TH: tyrosine hydroxylase
